# A model for the assessment of bluetongue virus serotype 1 persistence in Spain

**DOI:** 10.1371/journal.pone.0232534

**Published:** 2020-04-30

**Authors:** Cecilia Aguilar-Vega, Eduardo Fernández-Carrión, Javier Lucientes, José Manuel Sánchez-Vizcaíno

**Affiliations:** 1 VISAVET Health Surveillance Centre, Animal Health Department, Faculty of Veterinary Medicine, Complutense University of Madrid, Madrid, Spain; 2 Department of Animal Pathology (Animal Health), AgriFood Institute of Aragón IA2, Faculty of Veterinary Medicine, University of Zaragoza, Zaragoza, Spain; Faculty of Science, Ain Shams University (ASU), EGYPT

## Abstract

Bluetongue virus (BTV) is an arbovirus of ruminants that has been circulating in Europe continuously for more than two decades and has become endemic in some countries such as Spain. Spain is ideal for BTV epidemiological studies since BTV outbreaks from different sources and serotypes have occurred continuously there since 2000; BTV-1 has been reported there from 2007 to 2017. Here we develop a model for BTV-1 endemic scenario to estimate the risk of an area becoming endemic, as well as to identify the most influential factors for BTV-1 persistence. We created abundance maps at 1-km^2^ spatial resolution for the main vectors in Spain, *Culicoides imicola* and Obsoletus and Pulicaris complexes, by combining environmental satellite data with occurrence models and a random forest machine learning algorithm. The endemic model included vector abundance and host-related variables (farm density). The three most relevant variables in the endemic model were the abundance of *C*. *imicola* and Obsoletus complex and density of goat farms (AUC 0.86); this model suggests that BTV-1 is more likely to become endemic in central and southwestern regions of Spain. It only requires host- and vector-related variables to identify areas at greater risk of becoming endemic for bluetongue. Our results highlight the importance of suitable *Culicoides* spp. prediction maps for bluetongue epidemiological studies and decision-making about control and eradication measures.

## Introduction

Bluetongue is an infectious, non-contagious, arboviral disease that affects primarily ruminants, both domestic and wild, and whose biological vector are midges of the genus *Culicoides* [1]. Bluetongue is caused by bluetongue virus (BTV), which belongs to the genus *Orbivirus* [1]. To date, more than 30 BTV serotypes have been described [2]. Bluetongue is a listed disease of the World Organization of Animal Health because of its transboundary nature and major economic impact [3].

Prior to 1998, bluetongue was reported sporadically in Europe, but since then numerous BTV serotypes have been reported in the continent, including in Spain [4]. The first reported cases of bluetongue in Spain was caused by BTV-10 in 1956 [5]. After this was eradicated, no bluetongue outbreak was recorded until 2000, when BTV-2 emerged on the Balearic Islands. Since 2003, outbreaks of serotypes BTV-1, -4 and -8 from different sources have occurred in the country [6]. In 2007, BTV-1 was reported for the first time and was reintroduced in 2014 through the south [7]. At the beginning of the BTV-1 epizooty, the virus spread northward and reached the northern border of the country within a year [8]. This serotype caused mean mortality of 7% and higher morbidity than other serotypes such as BTV-4 [9]. Control programs against BTV-1, which included banning susceptible host movement to non-infected areas and vaccination within infected ones [10], drastically reduced reported outbreaks and clinical signs in susceptible hosts [11]. Since 2015, northern and eastern Spain have been considered free from BTV-1 [12]. In some other regions, however, BTV-1 remains endemic: cases have been reported annually since 2015, although not in 2018 [13]. The history of BTV in Spain and its continuing endemism make the country an excellent model for understanding BTV epidemiology.

The present study aimed to develop a model for understanding BTV-1 epidemiology in Spain under an endemic scenario. The model was based on abundance maps of *Culicoides* spp., whose blood-feeding serves as the epidemiologically only relevant route of spread for BTV serotypes 1–24 [14], although other transmission routes have been described for novel serotypes [15, 16]. *Culicoides imicola* is considered the major BTV vector in Africa, Middle East, Southeast Asia and Southern Europe [14]. Palearctic species are also competent vectors in Europe, such as *Culicoides chiopterus*, *Culicoides dewulfi*, and the Obsoletus and Pulicaris complexes, which explained the appearance of BTV in areas lacking *C*. *imicola* [17].

We adopted a different approach from previous BTV epidemiological studies when generating *Culicoides* spp. abundance maps. Previous studies estimated *Culicoides* spp. using inverse distance-weighted interpolation [18] or Poisson regression based on previously identified correlation of Obsoletus complex catches with temperature and precipitation [19]. In the present study, *Culicoides* spp. abundance maps were generated using a machine learning method.

The goal of our analysis was to estimate the risk that an area would become endemic using only host and vector variables, to understand the factors most relevant for the persistence of BTV-1 in an endemic scenario, and to describe the spatio-temporal evolution of BTV-1 in Spain.

## Materials and methods

### Predicting the probability of occurrence and abundance of *Culicoides* spp. in Spain

#### Entomological data and predictive variables

**Fig 1:**
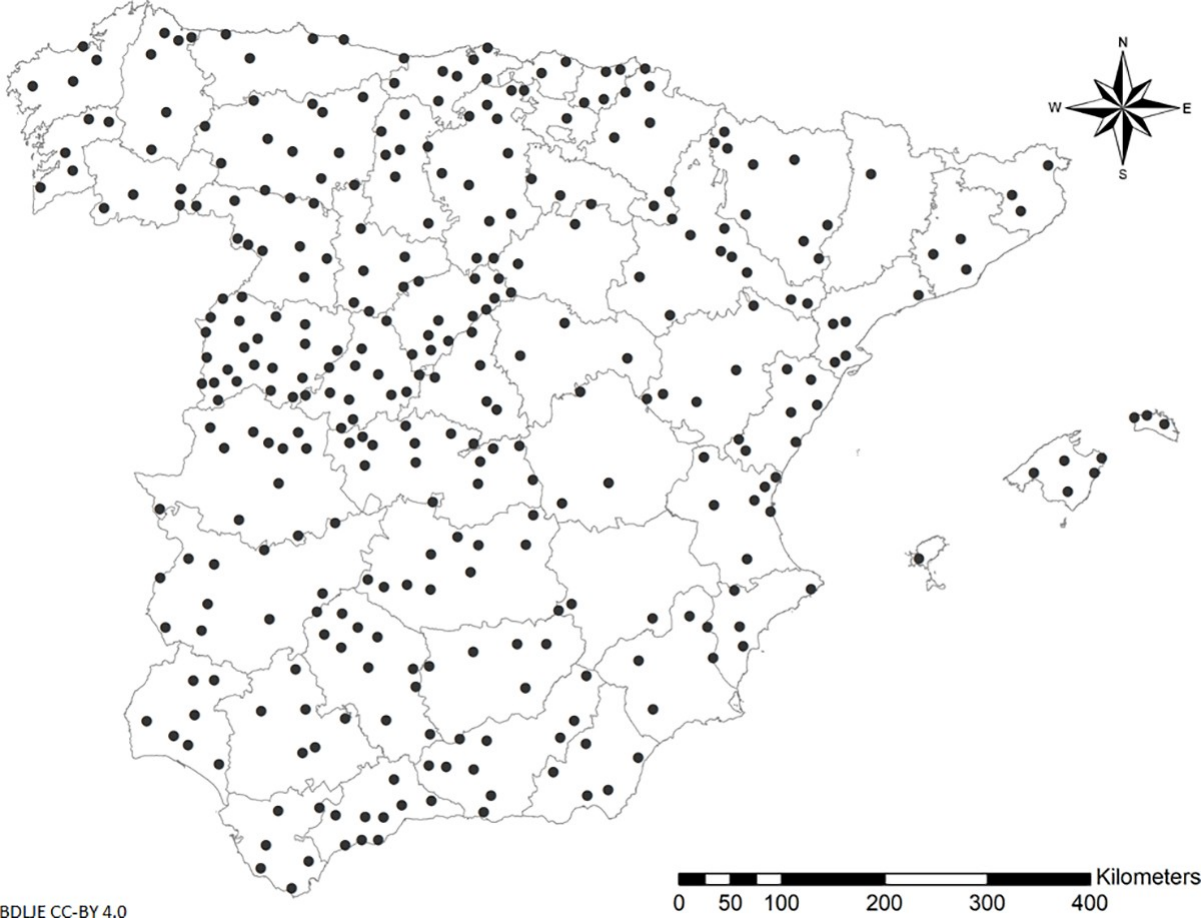
Locations of *Culicoides* spp. trap sites in mainland Spain and the Balearic Islands in the period 2005–2015. Administrative boundaries provided by Instituto Geográfico Nacional (IGN); BDDAE CC-BY 4.0.

Data on *Culicoides* spp. catches used in this study were collected in mainland Spain and the Balearic Islands from 2005 to 2015 as part of the Bluetongue National Surveillance Program. Details of catch methodology [20, 21] and species identification [22] have been described elsewhere. Miniature CDC UV-light traps were placed once a week, from dusk until dawn, in the same municipality close to animal holdings [21]. We used data for *C*. *imicola*, the Obsoletus complex (*Culicoides obsoletus* and *Culicoides scoticus*) and the Pulicaris complex (*Culicoides pulicaris* and *Culicoides lupicaris*). Data on species within each complex were aggregated because of the difficulty of differentiating females of the species macroscopically [22]. Data were analyzed for the period from April to October as in previous studies [22–24], when midge activity is higher and *Culicoides* species populations in Spain show a peak [25]. At each site with at least one catch per month during the period of study, we determined maximum abundance for each *Culicoides* spp. per year; the results are likely to be representative of the real annual *Culicoides* spp. population abundance [26]. In order to avoid spatial autocorrelation, locations closer than 10 km were excluded for further analysis. In the end, 331 trap sites (**Fig 1**) and 992 observations were selected for *Culicoides* spp. models.

*Culicoides* spp. occurrence was assessed as a function of 21 variables related to climate, vegetation indexes, host, orography, land cover and soil type (**Table 1**). Climatic variables are closely related to arthropod lifecycle and distribution [27, 28]. Within vegetation indexes, NDVI has been proved to be a proxy of biomass vegetation and phenology as well as other foliar parameters, and it correlates with soil moisture [29]. EVI was developed to enhance NDVI limitations by minimizing aerosol effects and improving the sensitivity in areas with high biomass conditions [30], whilst MIR band is used in some vegetation studies as complementary to vegetation indexes [31]. Host availability is necessary for the presence of hematophagous biting midges since blood feeding is required for oviposition [28]. The species studied here feed mainly on mammalian hosts, which includes ruminant species sensible to BTV [32, 33]. Altitude can be used as an indirect indicator of temperature, rainfall and solar radiation [34], while land cover and soil type are related to *Culicoides* spp. breeding habitat suitability [35, 36].

**Table 1:** Variables included in the models of *Culicoides* spp. occurrence.

Category	Variable description	Units	Spatial resolution	Temporal resolution	Data origin
Vegetation indexes	Mean Normalized Vegetation Index (NDVI)	-	250 x 250 m	16 days	National Aeronautics and Space Administration’s (NASA) Moderate Resolution Imaging Spectroradiometer product MOD13Q1 [38], downloaded from https://ladsweb.nascom.nasa.gov/
	Mean Enhanced Vegetation Index (EVI)				
	Mean medium-infrared reflectance (MIR)				
Climatic	Mean day-time surface temperature (LSTd)	°C	1 km^2^	8 days	NASA’s Moderate Resolution Imaging Spectroradiometer product MOD11A2 [39], downloaded from https://ladsweb.nascom.nasa.gov/
	Mean night-time surface temperature (LSTn)				
	Mean precipitation	mm	~1 km^2^	Monthly	WorldClim dataset (www.worldclim.org) [40]
	Mean wind speed	m/s			
Hosts	Livestock density (sheep, cattle and goat)	animals/km^2^	1 km^2^	-	Gridded Livestock of the World v2.0 [41]
	Probability of presence of red deer	%	~1 km^2^	-	[42]
Orography	Altitude	meters	~1 km^2^	-	Global 30 Arc-Second Elevation available at the U.S.G.S. [43]
Land cover / Land use	Rainfed cropland (LC1)	%	300 x 300 m	Annual	Climate Change Initiative Land Cover from the European Space Agency [44]
	Irrigated cropland (LC2)				
	Mix of cropland and natural vegetation (LC3)				
	Broadleaved tree cover (LC4)				
	Mix of tree/shrub cover and grassland (LC5)				
	Grassland (LC6)				
	Urban areas (LC7)				
Soil type	Clay	%	500 x 500 m	-	Topsoil physical properties for Europe [45] available at the European Soil Data Centre (https://esdac.jrc.ec.europa.eu/)
	Sand				
	Silt				
	Topsoil organic carbon content (OCTOP)	%	1 km^2^	-	Topsoil Organic Carbon Content for Europe [46] available at: https://esdac.jrc.ec.europa.eu/

The probability of occurrence of each midge species was added as a new variable to the abundance model [37]. For variables measured continuously throughout the year (vegetation indexes and temperature), data from 2005 to 2015 was retrieved, and the mean value for April-October of each year was obtained. All variable maps were transformed to adjust to the same extent and 1-km^2^ spatial resolution and projected onto the same coordinate system using ArcMap^TM^ v10.4.1. (Esri^®^). Variable values were extracted for each site, and for the percentage of land cover/use a 500-m radius buffer at each trapping site was used in ArcMap. Correlations among variables were explored using Spearman correlation analysis.

#### Data preprocessing and model selection

The endemic BTV-1 model was built using *Culicoides* spp. abundance instead of probability of occurrence, since these two variables show a non-linear relationship [37], and abundance is more relevant to study epidemiological processes [47]. Nevertheless, probability of occurrence maps were generated to serve as a predictive variable in *Culicoides* spp. abundance maps [37]. The number of catches (*C*) of the *C*. *imicola* and the Obsoletus and Pulicaris complexes were transformed into presence and absence classes. Since catch data were imbalanced in some of the datasets, we applied an over- and under-sampling technique to the training dataset using the R package “DMwR” [48], in order to improve model performance [49, 50]. The Synthetic Minority Over-sampling Technique (SMOTE) algorithm over-samples the minority class, generating synthetic new observations between *k*-nearest neighbors, whereas it randomly under-samples the majority class [51]. For the abundance models, the number of *Culicoides* spp. catches per site was transformed to *log*_*10*_(*C*+1).

The best algorithm for both *Culicoides* spp. occurrence and abundance models was selected from various machine learning techniques using the Python module “scikit-learn” [52]. The following algorithms were tested for occurrence models: *k*-nearest neighbors for classification (KNN), AdaBoost for classification (ABC) and random forest for classification (RFC). The following algorithms were tested for abundance models: lasso regression (LASSO), KNN, AdaBoost for regression (ABR) and random forest for regression (RFR). Details of all these algorithms can be found elsewhere [53]. Datasets for *C*. *imicola* as well as Obsoletus and Pulicaris complexes were randomly split into a training dataset (70%) and test dataset (30%). Proven models were developed using 10-fold cross-validation in the training dataset. The best occurrence model was selected based on mean recall (proportion of real positives classified as such) and precision (proportion of true positives among predicted positives). The best abundance model was selected based on the mean square error (MSE) of the training dataset.

#### Abundance of *Culicoides* spp

Once the best algorithm was chosen for occurrence models, it was implemented for all *Culicoides* spp. occurrence models using the variables in **Table 1**. Occurrence model performance was assessed in terms of sensitivity, specificity (proportion of real negatives classified as such), and the area under the receiver operating characteristic curve (AUC) using the test dataset. The AUC measures how well the model discriminates between presence and absence locations [34]. Values of AUC range from 0 to 1, with 1 indicating perfect discriminatory power [54].

The best algorithm was chosen for abundance models using the variables gathered in **Table 1**, as well as including the probability of occurrence for the corresponding *Culicoides* spp. as a predictor [37]. The final abundance models were built in R software v3.5.2 [55], and the model’s predictive ability was assessed in terms of mean absolute error (MAE) and root mean squared error (RMSE) using the test dataset. While MAE weights all errors the same, RMSE weights outlier errors more [56]. Both estimators can range between 0 and ∞, and values closer to 0 indicate better fit of the regression model [56]. The absence of spatial autocorrelation for the residuals of RFR models was tested using Moran’s I test in ArcMap.

For 1-km^2^ prediction maps, mean annual raster maps were generated, and the “raster” package [57], was used to generate prediction maps from the fitted models.

### BTV-1 endemic model

#### Study period and variables for the endemic model

The Spanish Bluetongue National Surveillance Program involves vaccination, restriction of susceptible host movement, as well as passive clinical, entomological, serological and virological surveillance for the early detection of BTV circulation [6, 12]. Sentinel animals are tested periodically such that 5% prevalence can be detected with a 95% confidence interval in every province (the surveillance epidemiological unit) [12]. Sampling occurs at least monthly from May to December in defined risk areas, and maximally twice yearly in non-risk areas [12], with possible variation among years. Samples are analyzed using enzyme-linked immunosorbent assay, and positive samples are further analyzed using polymerase chain reaction and virus serotyping in the Spanish National Reference Laboratory [12].

11,486 BTV-1 outbreaks in livestock holdings from 2007 to 2017 were retrieved at municipality level from the Spanish Ministry of Agriculture, Fishery and Food [13]. An outbreak was defined as a BTV-infected farm with at least one positive animal confirmed by the National Reference Laboratory, regardless of whether animals exhibited clinical signs [58]. The outbreak date corresponds to the day of confirmation by the laboratory. Numbers of cattle, sheep and goat farms were retrieved at province level from the Spanish 2009 agrarian census [59], and transformed to farm density (farms/km^2^). Annual variation in farm densities was estimated from variation in the number of farms in 2009 at the level of Autonomous Community, based on the 2019 report of the Integral Animal Traceability System (SITRAN in Spanish), from the Spanish Ministry of Agriculture, Fishery and Food [13]. The annual farm density for each livestock species corrected with the annual variation was used as a variable for the model. Mean predicted abundances of *C*. *imicola* as well as Obsoletus and Pulicaris complexes were obtained for each province and used as variables.

The factors associated with BTV-1 persistence were analyzed at province level (the epidemiological unit of surveillance [60]), only for mainland Spain since BTV-1 has never been recorded on the Balearic Islands. BTV-1 outbreaks occurred 11 years from 2007–2017; no BTV-1 outbreak was reported in 2018 [13, 61]. Two different scenarios were defined, an endemic and epidemic one. We defined the epidemic scenario as the period when there was geographical expansion of the disease and viral circulation was high, and the endemic scenario as the period when no geographical expansion occurred and viral circulation was low, based on reported outbreaks. BTV-1 outbreaks expanded geographically in Spain from 2007 to 2009, so this period was defined as the epidemic scenario, leaving 2010–2017 as the endemic scenario. In this study we only studied the endemic scenario.

#### Statistical analysis of the endemic model

In an endemic scenario, outbreaks are infrequent events because of natural or vaccine-mediated immunization of the susceptible population, and because of the lack of clinical signs in many infected animals. Therefore, we transformed the annual number of outbreaks per province into binary data on presence or absence of viral circulation for each year in the period of 2010–2017, considering the lack of BTV-1 notification as absence. The full dataset was randomly split into training (70%) and test (30%) datasets, and the training dataset was balanced using the SMOTE algorithm [51]. We used the RFC algorithm to estimate the risk of BTV-1 persistence in mainland Spain using the training dataset.

The random forest (RF) algorithm grows decision trees, which aggregate to make a prediction. At each node in the process of growing trees, the variable that minimizes the impurity is selected for binary splitting from the variables randomly sampled as candidates at each node (*mtry*) [62]. The decrease in node impurities is measured and, when the RF model is complete, the average of these measures gives the importance of each variable in the model through the mean decrease Gini for classification and increase in node purity (INP) for regression. Higher values mean greater importance of the variable [63]. In addition, when a tree is built, approximately one-third of the dataset is not used, and this is the *out-of-bag* (OOB) data [62]. The OOB estimate or error rate is calculated after aggregating the OOB predictions, and it is a measure of the prediction error of the algorithm [63]. The models were developed in R software [55] using the libraries “randomForest” [63], “DMwR” [48], “caret” [64] and “pROC” [65]. RFC *mtry* was set in order to reduce the error rate of the models, and 500 trees were used. BTV-1 endemic model performance was assessed in terms of sensitivity, specificity and AUC using the test dataset. Variable importance was assessed using the mean decrease Gini.

In order to generate the BTV-1 endemism risk map with the “raster” package [57], we used rasterized maps showing the mean density of ruminant livestock farms and the maps of average abundance of *Culicoides* spp. by province. We used three risk categories whose cut-off was calculated using the natural break classification method [66].

## Results

### *Culicoides* spp. distribution models

#### Model selection and evaluation

**Fig 2:**
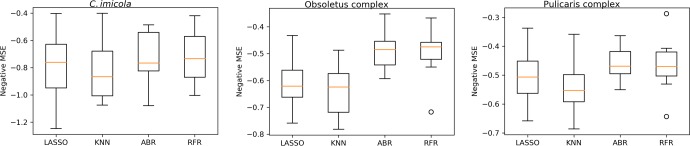
Comparison of machine learning algorithms for the models of *Culicoides* spp. abundance. Where LASSO: lasso regression; KNN: *k*-nearest neighbors for regression; ABR: AdaBoost for regression; RFR: random forest for regression.

After the balance of some training datasets with SMOTE (**S1 Table**), we selected RF as the algorithm to be used in all models since it performed best in general for all *Culicoides* species, although for some models it performed similarly to AdaBoost algorithm (**Table 2** and **Fig 2**). RFC is more balanced than the other algorithms when taking into account both recall and precision measures, which implies that RFC is good for identifying and predicting true positives. We applied RFC the same as for BTV-1 model; for abundance models, *mtry* was set in order to obtain the minimum MSE (**S1 Table**) and 500 trees were used for modeling. Since RF can deal with collinearity [62], all variables were included in the modeling. Variable importance was assessed in terms of the mean decrease Gini.

**Table 2:** Comparison of machine learning algorithms for the models of *Culicoides* spp. occurrence. Where, KNN: *k*-nearest neighbors for classification; ABC: AdaBoost for classification; RFC: random forest for classification. The mean and standard deviation (std) is provided for each measure.

Model	*C*. *imicola*		Obsoletus complex		Pulicaris complex	
	Recall (std)	Precision (std)	Recall (std)	Precision (std)	Recall (std)	Precision (std)
KNN	0.71 (0.07)	0.68 (0.1)	0.69 (0.09)	0.73 (0.1)	0.68 (0.12)	0.67 (0.1)
ABC	0.84 (0.1)	0.8 (0.09)	0.77 (0.06)	0.8 (0.1)	0.74 (0.08)	0.71 (0.11)
RFC	0.8 (0.09)	0.85 (0.07)	0.8 (0.07)	0.85 (0.09)	0.8 (0.08)	0.81 (0.09)

**Fig 3:**
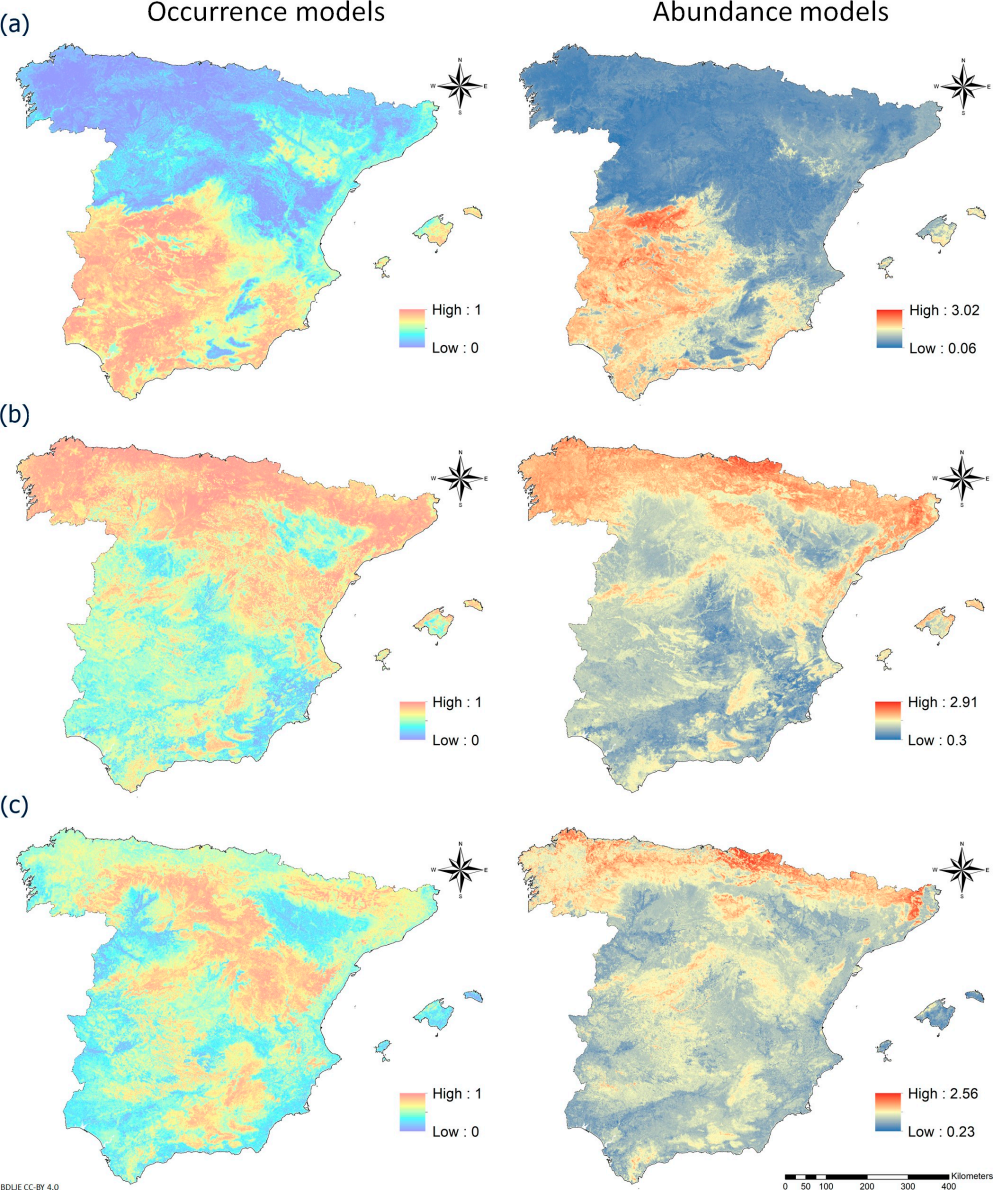
Maps of predicted occurrence and abundance of (a) *C*. *imicola*, (b) Obsoletus complex, or (c) Pulicaris complex in mainland Spain and the Balearic Islands generated using the random forest algorithm. Abundance is presented on a logarithmic scale [*log*_10_(*C* + 1), where *C* is the number of *Culicoides* spp.]. Administrative boundaries provided by Instituto Geográfico Nacional (IGN); BDDAE CC-BY 4.0.

According to occurrence and abundance models (**Fig 3**), *C*. *imicola* was the only species predicted to be absent anywhere: our maps showed it to be absent from northern areas. The predicted maximal densities of species varied substantially: the maximum for *C*. *imicola* was predicted to be 1,046.13 midges/km^2^, compared to 811.83 midges/km^2^ for Obsoletus complex or 362.08 midges/km^2^ for Pulicaris complex.

The occurrence model based on RFC showed better predictive and discriminatory ability for *C*. *imicola* (AUC 0.87), than for the Obsoletus (AUC 0.74) and Pulicaris (AUC 0.75) complexes (**Table 3**). The abundance model for Palearctic species appeared superior to the model for *C*. *imicola*, giving better MAE and RMSE.

**Table 3:** Performance of models of *Culicoides* spp. occurrence and abundance in Spain.

	Occurrence models			Abundance models	
	Sensitivity	Specificity	AUC^a^	MAE^b^	RMSE^c^
***C*. *imicola***	0.82	0.91	0.87	0.60	0.76
**Obsoletus complex**	0.76	0.72	0.74	0.52	0.64
**Pulicaris complex**	0.76	0.74	0.75	0.51	0.64

^a^AUC = area under the receiver operating characteristic curve

^b^MAE = mean absolute error

^c^RMSE = root mean squared error

#### Importance of different variables for *Culicoides* spp. occurrence and abundance

The most important variables according to the mean decrease Gini for RFC and the increase in node purity for RFR are shown in **S2 Table**, and correlations between variables are explored in **S1 Fig**. *C*. *imicola* occurrence was highly influenced by climatic variables (temperature and precipitation). Its distribution was also affected by vegetation indices, altitude, livestock density and some properties of the topsoil (OCTOP and silt contents). Similar to the case of *C*. *imicola*, the probability of Obsoletus complex occurrence was driven mainly by temperature, OCTOP and precipitation, but also by vegetation indices, and topsoil physical properties. The most significant variables in the case of Pulicaris complex were altitude and temperature. Probability of presence of red deer was also relevant, as were wind speed, OCTOP and topsoil physical properties.

The most meaningful predictor of abundance in all the *Culicoides* spp. models was the probability of occurrence of the corresponding species. *C*. *imicola* abundance was influenced heavily by climatic factors as well as livestock density, altitude and precipitation. Altitude and wind speed were more important than vegetation indices in the occurrence model of *C*. *imicola*. Abundance of Obsoletus complex was determined mainly by climatic factors, vegetation indices, OCTOP and altitude. Abundance of Pulicaris complex was influenced most by temperature, wind speed, vegetation indices, probability of red deer presence, precipitation and livestock density.

### BTV-1 endemic model

#### Spatio-temporal distribution of BTV-1 outbreaks and variables

**Fig 4:**
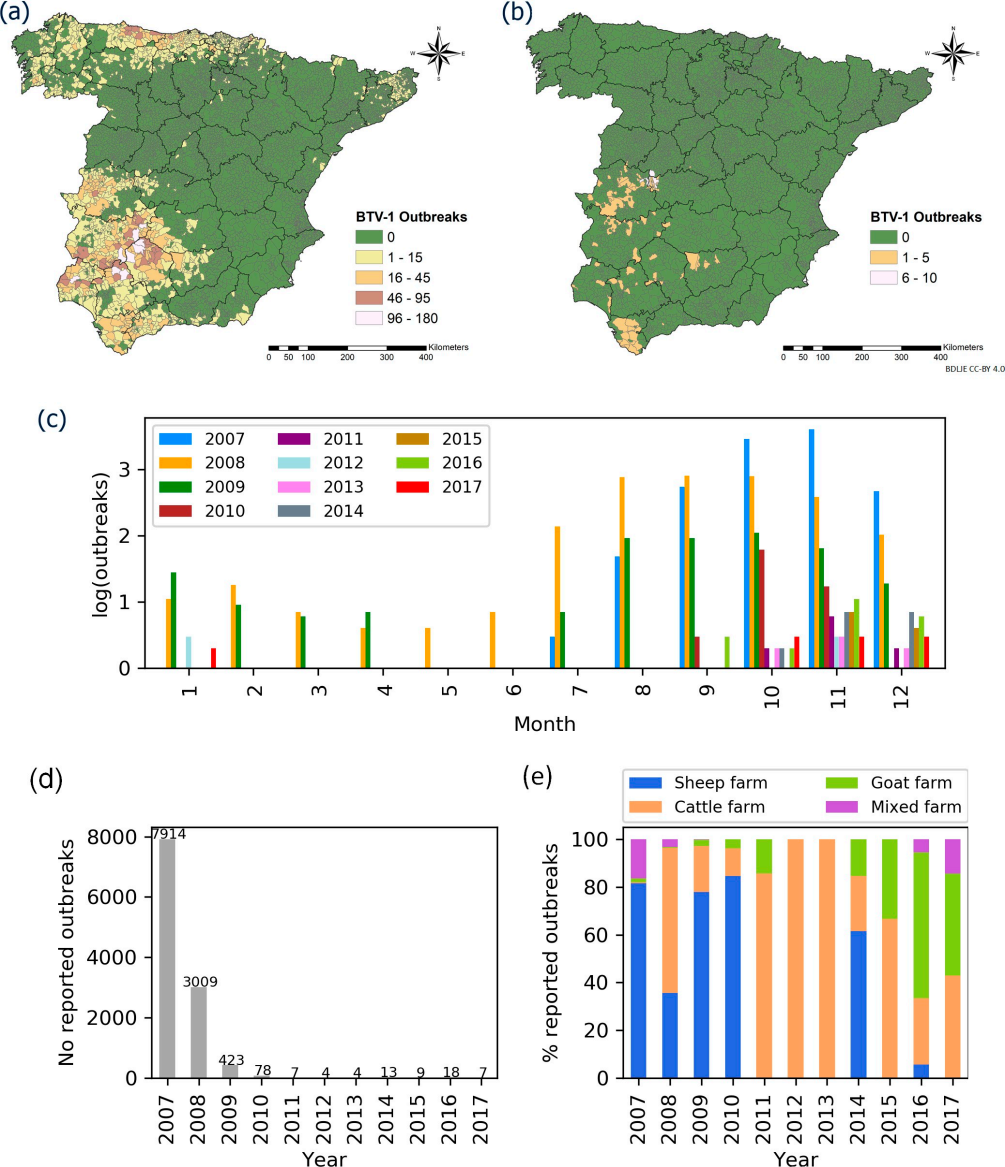
Number of reported BTV-1 livestock outbreaks per municipality in Spain under (a) the epidemic scenario (2007–2009) or (b) the endemic scenario (2010–2017). (c) Numbers of outbreaks, presented on a logarithmic scale [*log*_*10*_(*x*+1)], per month and year. (d) Number of reported outbreaks per year. (e) Percentages of different farm types affected in the outbreaks. Darker lines in the map delineate provinces. Administrative boundaries provided by Instituto Geográfico Nacional (IGN); BDDAE CC-BY 4.0.

A total of 11,486 BTV-1 outbreaks occurred in livestock holdings from 2007 to 2017, with different spatial distributions and frequencies depending on the scenario (**Fig 4A** and **4B**; **S3 Table**). The outbreaks showed strong seasonality, with more of them occurring in October and November (**Fig 4C**). However, differences between years were observed: in 2008 and 2009, outbreaks were reported every month, with notifications increasing in July and remaining fairly constant in August-October; in other years, notifications peaked in October-November. Nearly all reported outbreaks (98.78%) occurred in 2007–2009, supporting our assumption that outbreaks are infrequent in an endemic scenario (**Fig 4D**). Most outbreaks during the epidemic scenario (2007–2009) occurred on sheep farms (69.32%), followed by cattle farms (17.12%), mixed farms (12.33%) and goat farms (1.22%). The corresponding frequencies in the endemic scenario showed a redistribution across these farm types: 53.57%, 28.57%, 1.43% and 16.43% (**Fig 4E**). In 2007, most outbreaks were reported in southern and southwestern areas of mainland Spain; in 2008 and 2009, outbreaks tended to occur in northern regions and western and central areas (**S2 Fig**). In 2010, the spatial distribution of outbreaks was significantly reduced, being reported mainly in western and central areas; and during 2011–2013 notifications were limited to that zone. In 2014, no more outbreaks were reported there, but BTV-1 appeared in the South of mainland Spain after six years of absence. National authorities attributed this reappearance to a reintroduction of BTV-1 from Morocco, where the virus was circulating [7]. During subsequent years, outbreaks have been declared in southwestern areas until 2018, when no outbreak was recorded [61].

The distribution of farms around Spain depended on the species (**S3 Fig**). Cattle farms were denser in northwestern provinces, but also in northern and western provinces. Sheep farms were denser over more extensive regions of the country, though their density was highest in northwestern provinces. Goat farms were denser in southeastern and northwestern provinces. Numbers of cattle and sheep farms in Spain decreased from 2007 to 2012, after which they remained stable. The number of goat farms, in contrast, slowly increased from 2012.

#### Statistical analysis of the BTV-1 endemic scenario

**Fig 5:**
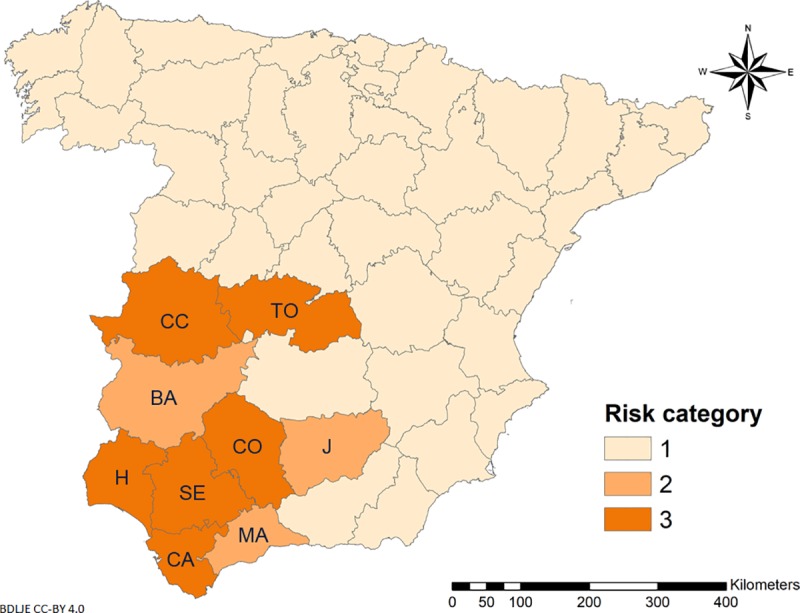
Risk of BTV-1 endemism in Spain (2010–2017), generated by random forest for classification (RFC) algorithm. Dark orange show areas at more risk of BTV-1 maintenance according to the risk categories generated by Jenks natural break classification method. BA: Badajoz; CC: Caceres; CA: Cadiz; CO: Cordoba; H: Huelva; J: Jaen; MA: Malaga; SE: Seville; TO: Toledo. Administrative boundaries provided by Instituto Geográfico Nacional (IGN); BDDAE CC-BY 4.0.

After applying the SMOTE algorithm to the training dataset, we obtained 170 positive observations from a total of 368 (46.20%), emanating from 17 (6.46%) positive observations of 263 total original observations. The BTV-1 endemic model (*mtry* = 2), which showed an AUC of 0.86, was good at identifying areas where BTV was absent (specificity = 0.97) but less reliable for identifying affected areas (sensitivity = 0.75). The most important variable contributing to the model was *C*. *imicola* abundance, followed by abundance of Obsoletus complex and density of goat farms (**Table 4**). The model was less influenced by density of cattle farms and abundance of Pulicaris complex. Our model suggests that BTV-1 is more likely to persist in central and southwestern Spain (**Fig 5**).

**Table 4:** Importance of variables in the BTV-1 endemic model based on the mean decrease Gini (MDG).

Variable	MDG
*C*. *imicola* abundance	55.22
Obsoletus complex abundance	31.91
Density of goat farms	30.83
Density of sheep farms	24.92
Density of cattle farms	21.03
Pulicaris complex abundance	16.38

## Discussion

### *Culicoides* spp. distribution models

In this study, we have developed distribution models for the most relevant bluetongue vectors in Spain. Predicted abundance and seasonality of insect vector species are useful tools to improve surveillance for new arbovirus introductions, as well as to assess their transmission, spread and persistence [47]. The tropical species *C*. *imicola* differed substantially from the Palearctic species Obsoletus and Pulicaris complexes in distribution and abundance (**Fig 3**), and in how well their distribution could be modeled (**Table 3**). Predicted *C*. *imicola* abundance is limited by the Central system (a mountain range located in central-western of the Iberian Peninsula). The Obsoletus complex is predicted to be more abundant in northern Spain and in the Iberic, Central and Betic systems, which feature cooler, wetter climates. Indeed, the Pulicaris complex is strongly associated with mountainous reliefs (**Fig 3**). The ubiquity of Palearctic species in Spain, depicted by the low number of negative catches in the datasets (**S1 Table**), may explain why our models were slightly less reliably predicting locations of absence, even after balancing positive and negative observations. Our results confirm the importance of assessing model performance using several parameters beyond only AUC [67]. The predictions of *Culicoides* spp. abundance presented here are a useful tool for risk assessment and decision-making for surveillance, control and eradication programs.

RF outperformed all the other algorithms for both the occurrence and abundance models. RF is a non-parametric technique that makes no *a priori* assumptions about the distributions of the dependent variables and it usually outperforms parametric techniques [24, 68]. It also indicates the importance of variables in the final model [62], although it does not indicate the *direction* of their importance, in contrast to parametric techniques. We did not require an indication of the direction of variables' importance because many studies using parametric techniques have identified factors that drive *Culicoides* spp. ecology and have described how those factors interact with *Culicoides* distribution or abundance [22, 23, 26, 69–74]. Although RF tolerates variable collinearity, some studies have shown that correlated variables can be favored in the process of growing trees [75]. However, variable importance estimated through the mean decrease Gini was shown to be capable of identifying relevant predictor variables in datasets with highly correlated variables [76]; and as discussed below, the identified relevant variables coincided with previous work.

Our distribution and abundance models clearly establish the importance of climatic variables for *Culicoides* spp. modeling (**S2 Table**). This supports the conclusions of several previous studies that included only climatic variables in the final models [69, 70, 72]. We believe that our approach is more accurate because climatic variables alone do not capture the complexity of *Culicoides* spp. habitat. Our study also found host distribution to be important for both occurrence and abundance models, consistent with the few models that include this variable [23, 77]. Presence of red deer was less important in *C*. *imicola* and the Obsoletus complex models than livestock density, which may reflect the fact that we used probability of presence instead of abundance, and the fact that *Culicoides* spp. trap sites are placed close to livestock holdings [20]. Our finding that wind speed contributes substantially to abundance models is consistent with reports of a negative correlation between wind speed and size of *Culicoides* spp. catches [73, 78].

*C*. *imicola* chooses breeding sites mainly on the basis of vegetation indices, organic carbon content and percentage of silt composition. This species breeds in rich organic matter and water-saturated soils [79]; it does not inhabit sandy soils, which drain quickly and offer sparse nutrients [80]. Consistent with these preferences, silt content in our *C*. *imicola* models correlated negatively with sand content (**S1 Fig**). The Obsoletus complex prefers breeding sites with high organic content [81], consistent with the importance of vegetation indices, OCTOP, clay and silt composition of the soil in our Obsoletus models. Breeding site preferences of Pulicaris complex are ill-defined and likely flexible [81]. Our finding that altitude is quite important for Pulicaris occurrence is consistent with previous work [37, 71], although few studies have examined this complex on the Iberian Peninsula [37, 74]. Our results suggest that altitude should be taken into account when modeling Pulicaris complex distribution. Further work should identify additional determinants of distribution.

In general, vegetation indices and soil type were stronger predictors of *Culicoides* spp. distribution than land cover variables. Similarly, other *C*. *imicola* distribution models suggest a weak contribution by land cover or landscape variables [23, 82]. This may be a problem of insufficient data resolution: even the CORINE land cover index, a dataset of finer spatial resolution than the Climate Change Initiative Land Cover used here, cannot accurately predict *Culicoides* spp. breeding sites [83]. Thus, high-resolution remote sensing might be required to improve the performance of land cover variables for *Culicoides* spp. modeling.

The temporal resolution of the study (April to October) can imply some limitations especially for the Obsoletus complex, due to its greater abundance in comparison with the other species in the absent months [21]. During the excluded months, BTV-1 infection occurred several years (**Fig 4C**), demonstrating the possibility of BTV transmission during this period in a situation of high viral circulation. Despite being a plausible limitation to take into consideration, we excluded those months due to the decrease in sampling and positive catches register (**S4 Fig**). Likewise, the abundance peak for all the species is included in the period of study [25], making this limitation minor for maximal abundance models.

### BTV-1 endemic model

When a disease is introduced in a susceptible naïve population, it can spread rapidly if conditions favor transmission. Most BTV-1 outbreaks were reported in early years of the study period (**Fig 4D**), when sheep were the most clinically affected host [9, 84]. Outbreaks showed a marked seasonality (**Fig 4C**), with peaks occurring in November (2007 and 2011–2016), followed by October (2009–2010) and September (2008). Since outbreak dates correspond to when infection was confirmed in the laboratory, the infections giving rise to the outbreaks may have occurred approximately 1.5 months earlier [18]; the interval between infection and outbreak may depend on whether clinical signs are present and on whether sentinel holdings are affected. Thus, the period between infection and confirmation may have varied from year to year during the study period. In any case, infection rates are likely to be higher when *C*. *imicola* is more abundant (**S4 Fig**) in the endemic scenario (**Table 4**).

Our findings provide evidence that *C*. *imicola* abundance is of great importance for the persistence of BTV-1 (**Table 4**). These results reaffirm the crucial role of *C*. *imicola* in Spain and the Mediterranean Basin [14]; indeed, the low abundance of *C*. *imicola* in northern Spain may have favored the eradication of BTV-1 by vaccination [12]. At the same time, our findings suggest that the maintenance of BTV-1 is even more likely in areas where *C*. *imicola* and Palearctic species (in particular the Obsoletus complex) co-exist than in areas containing only *C*. *imicola*. The smaller significance of the Pulicaris complex in the model is consistent with studies suggesting that their biting rates are lower than for other *Culicoides* species [78, 85].

Other studies have previously highlighted the importance of livestock density for BTV presence [86, 87] and spread [88–90], but few included farm density in their final models [86, 88]. We found goat farms to influence BTV-1 maintenance more than other types of livestock farms in the endemic model, consistent with previous work [91]. In contrast, Pascual-Linaza *et al*. [18] did not found an association between BTV-1 occurrence and the number of goats in mixed farms in any of the performed models, although the spatial scale differs from ours. In a recent work [90], they determined cattle and sheep densities to be key for BTV spread and in lesser extent goat density. Our results may reflect, at least in part, the distribution of livestock and their farms in Spain (**S3 Fig**): goats and their farms are more abundant in southern regions, while cattle are more abundant in northern but also in western areas. Thus, our results might be partially revealing host availability in terms of farm density, however, they could also be indicating differences in management practices of the different farm systems, such as intensive *versus* extensive practices, biosecurity level of farms, vector control and turnover ratios, among others. In addition, goat vaccination has never been compulsory in Spanish regulation (APA/385/2019). Our model gives less importance as a variable to the density of cattle farms without specifying the *direction* of the association between the variable and the dependent variable due to the nature of the algorithm [61]. However other studies have defined a positive association between cattle density and BTV presence or spread [18, 86, 88]. In a work conducted in Spain in 2004–2005, they identified a negative association between cattle farm density and BTV-4 presence, and a positive association with small ruminant farm density and cattle density [86]. Cattle density in the proximity of sheep farms was identified as a risk factor for BTV-1 in late years of a study conducted in the Spanish region of Extremadura in 2007–2011 [18]. Cattle is widely considered as a major reservoir of BTV due to its prolonged viremia [92], therefore, our results should be taken into consideration with caution since they could be revealing anthropogenic factors associated with the ruminant livestock systems rather than the biological role of the different species. Moreover, the southern provinces at greater risk of BTV-1 persistence (**Fig 5**), correspond to the southern provinces with more density of cattle farms (**S3 Fig**). Thus, our results justify further and finer spatial scale analysis of the role of livestock, in particular goats, in BTV epidemiology to help guide BTV control and eradication programs. Ruminant wildlife species were not included for modeling. Viremia can persist in red deer for a long time, helping them to act as a reservoir [93], and the *Culicoides* spp. modeled in the present study have been identified in areas inhabited by wild ruminants [94]. Nevertheless, a virological and serological longitudinal monitoring of red deer from 2008 to 2015 performed in France showed that this species did not contribute to BTV spread or maintenance [95].

To control and eradicate BTV, Spain has always relied on vaccination as well as restriction of susceptible host movement from infected to non-infected areas [6]. Vaccination is key to the control and eradication of BTV [10], however, we could not assess its importance in the endemic scenario because the Spanish regulation ARM/1614/2011 made vaccination voluntary for BTV-1in restricted zones after June 30, 2011. As a result, owners of susceptible animals had to cover vaccination costs [60], leading to a significant drop in vaccination coverage [96]. Before June 30, 2011, an 80% of vaccination coverage was exceeded in susceptible animals in BTV-1 affected areas [12], although it was not homogeneously distributed [96]. These vaccination campaigns lead to a significant decrease in outbreaks (**Fig 4D**). In 2013, vaccination was obligatory in central-western areas where the virus was circulating. When BTV-1 was reintroduced in 2014 in southern Spain, an emergency vaccination program was established in the affected area since the immunity of susceptible population was low in the area [12], due to the low vaccination coverage in previous years along with the turnover ratio of the different ruminant species. During the period of voluntary vaccination (from July 2011 until 2014), less than a 20% of vaccination coverage was reached in voluntary vaccination areas [96]. In 2015, the Spanish regulation AAA/1424/2015 extended the area of compulsory vaccination, leading to an increase in vaccination coverage [96]. Vaccination campaigns after 2015 could have limited the spread of BTV-1 to central-western areas. After these vaccination campaigns, no BTV-1 outbreaks had been declared in 2018 nor in 2019 [13]. An 80% of effective vaccination coverage of susceptible domestic animals is needed to reduce the probability that the number of secondary cases exceeded or equaled primary cases [97]. Other work supported that even a 95% vaccination coverage of livestock for five years is not enough to avoid a re-emergence of BTV in Spain [98]. Thus, it can explain why circulation of BTV-1 was found in some areas of mainland Spain even when compulsory vaccination was implemented there [12].

Southwestern and central Spain appear to be at greater risk of BTV-1 persistence, consistent with previous work [18]. The risk of BTV-1 endemism of the Toledo province (**Fig 5**), should be analyzed with caution since outbreaks were only reported in western areas of this province (**Fig 4B**). In our study, the area at greater risk of BTV-1 maintenance is close to an area previously identified as being at greater risk of BTV-4 maintenance [86]. This underscores the importance of the distribution of BTV-1 and -4 vectors, in particular *C*. *imicola*, since it is the most abundant species in the area (**Fig 3**). Southern areas of Spain possess suitable conditions for BTV-1 circulation: *C*. *imicola*, Palearctic midges, sheep, goat and cattle are abundant. However, other factors could be favoring the persistence of BTV-1 in those areas; thus a more thorough study of the associated factors that also contributed to BTV-1 persistence should be conducted for all Spain. The possibility of BTV (re)introduction by wind currents should also be considered, as already documented in southern European countries such as Spain [99, 100], Portugal [101], and Italy [102]. The risk of windborne transportation of BTV-infected midges can be included in surveillance efforts to make them more cost-effective [99]. This requires active surveillance of nearby countries and effective communication among their governments.

Our model is not without limitations. First of all, it does not address the seasonality of BTV epidemiology in combination with *Culicoides* spp. seasonality, due to lack of data about the day of clinical suspicion or sampling. However, during the endemic scenario low number of outbreaks were reported, and mainly during October-December. A monthly analysis should be crucial for the epidemic scenario, in which many outbreaks are reported almost every month. Second, we did not include control measures, such as vaccination, as a variable in the model. Third, we did not assess the role of wild ruminant populations in BTV-1 epidemiology. We assumed that year-to-year variation in farm density was uniform within each Autonomous Community, which may not be accurate. Finally, we did not include BTV-4 in this study so we could not compare the risk of persistence of the two serotypes historically most relevant for Spain.

## Conclusion

Spain has experienced multiple infections of different BTV serotypes, which makes the country suitable for assessing risk factors for epidemic and endemic scenarios. We found that combining host data (farm density) with vector abundance predictions was sufficient to identify areas at greater risk of becoming endemic, providing a rapid and less data-demanding tool for bluetongue epidemiology. Our endemic model may be applicable to similar eco-climatic regions, i.e. Mediterranean Basin areas, for BTV serotypes for which *C*. *imicola* is considered the most competent vector [14]. Our findings (**Fig 5**) are a preliminary attempt to highlight the specific regions where BTV-1 persistence is high. They illustrate that reliable maps of *Culicoides* spp. abundance can contribute to a better understanding of bluetongue epidemiology and improve decision-making. Future studies should examine at-risk areas in finer temporal and spatial resolution including other factors that may affect virus maintenance, as reported for smaller regions of Spain [18], as well as assess the role of ruminant livestock in BTV epidemiology.

## Supporting information

S1 Table Calibration of the *Culicoides* spp. occurrence and abundance models. For occurrence models, it is shown numbers and percentages of *Culicoides* spp. catches in Spain used in the study as well as in the training dataset after application of the SMOTE algorithm.
(DOCX)

S2 Table Variable importance through the mean decrease Gini (MDG) and the increase in node purity (INP) for the *Culicoides* spp. occurrence and abundance models in Spain. (DOCX)

S3 Table Number of reported outbreaks per province and year. (DOCX)

S1 Fig Spearman correlation matrix for the variables used in the general *Culicoides* spp. models excluding the probability of occurrence acquired from the *Culicoides* spp. occurrence maps. (TIF)

S2 Fig Annual spatial distribution of BTV-1 outbreaks at municipality level from 2007–2017. Darker lines in the map delineate provinces. Administrative boundaries provided by Instituto Geográfico Nacional (IGN); BDDAE CC-BY 4.0.
(TIF)

S3 Fig Mean density of cattle, sheep and goat farms at province level form 2010–2017. Graphs below represent the mean percentage of variation of the number of farms in Spain on the basis of the 2009 density of livestock farms; vertical lines represent the standard deviation. Administrative boundaries provided by Instituto Geográfico Nacional (IGN); BDDAE CC-BY 4.0.
(TIF)

S4 Fig Mean *Culicoides* spp. seasonality (mean number of caught individuals) and percentage of positive catches from 2005–2015 in Spain. (TIF)
