# Supplementary material for: A model for the assessment of bluetongue virus serotype 1 persistence in Spain
Source: PLoS One. 2020 Apr 30;15(4):e0232534. doi: 10.1371/journal.pone.0232534 (PMC7192634; doi:10.1371/journal.pone.0232534)
Supplement: S1 Table — For occurrence models, it is shown numbers and percentages of Culicoides spp. catches in Spain used in the study as well as in the training dataset after application of the SMOTE algorithm. (DOCX) [file pone.0232534.s001.docx]

| **Species** | **Occurrence models** | | | | | **Abundance models** |
| --- | --- | --- | --- | --- | --- | --- |
|  | Initial number of *Culicoides* spp. catches | | Number of *Culicoides* spp. catches after SMOTE in the training dataset | | *mtry* | *mtry* |
|  | Positive catches/Total | % (SE %) | Positive catches/Total | % (SE %) |  |  |
| *C. imicola* | 454/992 | 0.46 (0.02) | 313/694* | 0.45 (0.02) | 3 | 7 |
| Obsoletus complex | 850/992 | 0.86 (0.01) | 309/618 | 0.5 (0.02) | 5 | 2 |
| Pulicaris complex | 662/992 | 0.67 (0.01) | 321/642 | 0.5 (0.02) | 8 | 10 |

^a^*mtry*: variables randomly sampled as candidates at each node

*****SMOTE algorithm was not applied
