# Supplementary material for: A model for the assessment of bluetongue virus serotype 1 persistence in Spain
Source: PLoS One. 2020 Apr 30;15(4):e0232534. doi: 10.1371/journal.pone.0232534 (PMC7192634; doi:10.1371/journal.pone.0232534)
Supplement: S2 Table — (DOCX) [file pone.0232534.s002.docx]

|  |  |  | 1 | 2 | 3 | 4 | 5 | 6 | 7 | 8 | 9 | 10 |
| --- | --- | --- | --- | --- | --- | --- | --- | --- | --- | --- | --- | --- |
| **Occurrence models** | *C. imicola* | Variable | LSTn | PREC | LSTd | OCTOP | ALT | LD | MIR | NDVI | SILT | EVI |
|  |  | MDG | 62.99 | 44.47 | 38.64 | 21.04 | 18.43 | 16.06 | 14.82 | 14.20 | 13.97 | 13.57 |
|  | Obsoletus complex | Variable | LSTd | OCTOP | PREC | NDVI | EVI | MIR | LSTn | SILT | SAND | CLAY |
|  |  | MDG | 38.26 | 29.99 | 25.62 | 18.36 | 17.61 | 17.60 | 17.22 | 15.67 | 15.10 | 14.98 |
|  | Pulicaris complex | Variable | ALT | LSTn | RD | WS | LSTd | OCTOP | SAND | SILT | CLAY | EVI |
|  |  | MDG | 30.11 | 28.70 | 20.90 | 19.75 | 18.84 | 18.35 | 17.76 | 16.85 | 16.45 | 15.79 |
| **Abundance models** | *C. imicola* | Variable | PO_IMIC | LSTn | LD | ALT | PREC | WS | LSTd | LC1 | SAND | OCTOP |
|  |  | MDG | 71.34 | 31.71 | 21.88 | 20.61 | 19.62 | 15.37 | 13.80 | 11.94 | 11.51 | 10.61 |
|  | Obsoletus complex | Variable | PO_OBSOL | PREC | LSTd | NDVI | MIR | EVI | LSTn | OCTOP | ALT | CLAY |
|  |  | MDG | 33.91 | 29.53 | 28.51 | 26.48 | 23.10 | 21.57 | 19.21 | 14.68 | 14.66 | 14.18 |
|  | Pulicaris complex | Variable | PO_PULIC | LSTd | LSTn | WS | NDVI | RD | MIR | LD | PREC | EVI |
|  |  | MDG | 21.01 | 16.77 | 14.34 | 13.18 | 12.74 | 11.00 | 10.92 | 10.37 | 10.31 | 9.84 |

NDVI: Mean Normalized Vegetation Index

EVI: Mean Enhanced Vegetation Index

MIR: Mean medium-infrared reflectance

LSTd: Mean day-time surface temperature

LSTn: Mean night-time surface temperature

PREC: Mean precipitation

WS: Mean wind speed

LD: Livestock density (sheep, cattle and goat)

RD: Probability of presence of red deer

OCTOP: Topsoil organic carbon content

LC1: Rainfed cropland

LC2: Irrigated cropland

LC3: Mix of cropland and natural vegetation

LC4: Broadleaved tree cover

LC5: Mix of tree/shrub cover and grassland

LC6: Grassland

LC7: Urban areas

PO_IMIC: Probability of occurrence of *Culicoides imicola*

PO_OBSOL: Probability of occurrence of the Obsoletus complex

PO_PULIC: Probability of occurrence of the Pulicaris complex
