# Supplementary material for: A model for the assessment of bluetongue virus serotype 1 persistence in Spain
Source: PLoS One. 2020 Apr 30;15(4):e0232534. doi: 10.1371/journal.pone.0232534 (PMC7192634; doi:10.1371/journal.pone.0232534)
Supplement: S3 Table — (DOCX) [file pone.0232534.s003.docx]

| **Autonomous Community** | **Province** | **2007** | **2008** | **2009** | **2010** | **2011** | **2012** | **2013** | **2014** | **2015** | **2016** | **2017** |
| --- | --- | --- | --- | --- | --- | --- | --- | --- | --- | --- | --- | --- |
| Andalusia | Almería | 25 | 0 | 0 | 0 | 0 | 0 | 0 | 0 | 0 | 0 | 0 |
|  | Cádiz | 448 | 1 | 0 | 0 | 0 | 0 | 0 | 13 | 1 | 0 | 4 |
|  | Córdoba | 1470 | 0 | 0 | 0 | 0 | 0 | 0 | 0 | 4 | 3 | 0 |
|  | Granada | 18 | 0 | 0 | 0 | 0 | 0 | 0 | 0 | 0 | 0 | 0 |
|  | Huelva | 1013 | 9 | 0 | 0 | 0 | 0 | 0 | 0 | 3 | 1 | 0 |
|  | Jaén | 99 | 0 | 0 | 2 | 0 | 0 | 0 | 0 | 0 | 2 | 2 |
|  | Málaga | 466 | 0 | 0 | 0 | 0 | 0 | 0 | 0 | 0 | 1 | 0 |
|  | Seville | 897 | 0 | 0 | 0 | 0 | 0 | 0 | 0 | 1 | 3 | 1 |
| Aragon | Huesca | 0 | 4 | 0 | 0 | 0 | 0 | 0 | 0 | 0 | 0 | 0 |
|  | Teruel | 0 | 0 | 0 | 0 | 0 | 0 | 0 | 0 | 0 | 0 | 0 |
|  | Zaragoza | 0 | 0 | 0 | 0 | 0 | 0 | 0 | 0 | 0 | 0 | 0 |
| Cantabria | Cantabria | 0 | 936 | 13 | 0 | 0 | 0 | 0 | 0 | 0 | 0 | 0 |
| Castilla-La Mancha | Albacete | 0 | 0 | 0 | 0 | 0 | 0 | 0 | 0 | 0 | 0 | 0 |
|  | Ciudad Real | 306 | 0 | 0 | 0 | 0 | 0 | 0 | 0 | 0 | 1 | 0 |
|  | Cuenca | 0 | 0 | 0 | 0 | 0 | 0 | 0 | 0 | 0 | 0 | 0 |
|  | Guadalajara | 0 | 0 | 0 | 0 | 0 | 0 | 0 | 0 | 0 | 0 | 0 |
|  | Toledo | 24 | 0 | 4 | 24 | 0 | 0 | 1 | 0 | 0 | 0 | 0 |
| Castile y León | Ávila | 0 | 0 | 3 | 10 | 0 | 0 | 0 | 0 | 0 | 0 | 0 |
|  | Burgos | 0 | 26 | 0 | 0 | 0 | 0 | 0 | 0 | 0 | 0 | 0 |
|  | León | 0 | 79 | 16 | 0 | 0 | 0 | 0 | 0 | 0 | 0 | 0 |
|  | Palencia | 0 | 52 | 0 | 0 | 0 | 0 | 0 | 0 | 0 | 0 | 0 |
|  | Salamanca | 0 | 0 | 5 | 0 | 1 | 0 | 0 | 0 | 0 | 0 | 0 |
|  | Segovia | 0 | 0 | 1 | 0 | 0 | 0 | 0 | 0 | 0 | 0 | 0 |
|  | Soria | 0 | 3 | 1 | 0 | 0 | 0 | 0 | 0 | 0 | 0 | 0 |
|  | Valladolid | 0 | 0 | 0 | 0 | 0 | 0 | 0 | 0 | 0 | 0 | 0 |
|  | Zamora | 0 | 0 | 11 | 0 | 0 | 0 | 0 | 0 | 0 | 0 | 0 |
| Catalonia | Barcelona | 0 | 24 | 9 | 0 | 0 | 0 | 0 | 0 | 0 | 0 | 0 |
|  | Gerona | 0 | 94 | 13 | 0 | 0 | 0 | 0 | 0 | 0 | 0 | 0 |
|  | Lérida | 0 | 7 | 1 | 0 | 0 | 0 | 0 | 0 | 0 | 0 | 0 |
|  | Tarragona | 0 | 3 | 1 | 0 | 0 | 0 | 0 | 0 | 0 | 0 | 0 |
| Community of Madrid | Madrid | 1 | 0 | 2 | 1 | 0 | 0 | 0 | 0 | 0 | 0 | 0 |
| Navarre | Navarre | 8 | 33 | 0 | 0 | 0 | 0 | 0 | 0 | 0 | 0 | 0 |
| Valencian Community | Alicante | 0 | 0 | 0 | 0 | 0 | 0 | 0 | 0 | 0 | 0 | 0 |
|  | Castellón | 0 | 2 | 2 | 0 | 0 | 0 | 0 | 0 | 0 | 0 | 0 |
|  | Valencia | 0 | 0 | 0 | 0 | 0 | 0 | 0 | 0 | 0 | 0 | 0 |
| Extremadura | Badajoz | 2235 | 34 | 5 | 0 | 0 | 0 | 0 | 0 | 0 | 7 | 0 |
|  | Cáceres | 843 | 119 | 84 | 41 | 6 | 4 | 3 | 0 | 0 | 0 | 0 |
| Galicia | A Coruña | 0 | 33 | 14 | 0 | 0 | 0 | 0 | 0 | 0 | 0 | 0 |
|  | Lugo | 0 | 185 | 7 | 0 | 0 | 0 | 0 | 0 | 0 | 0 | 0 |
|  | Ourense | 0 | 5 | 75 | 0 | 0 | 0 | 0 | 0 | 0 | 0 | 0 |
|  | Pontevedra | 0 | 3 | 148 | 0 | 0 | 0 | 0 | 0 | 0 | 0 | 0 |
| La Rioja | La Rioja | 0 | 14 | 1 | 0 | 0 | 0 | 0 | 0 | 0 | 0 | 0 |
| Basque Country | Álava | 0 | 87 | 6 | 0 | 0 | 0 | 0 | 0 | 0 | 0 | 0 |
|  | Guipúzcoa | 61 | 175 | 1 | 0 | 0 | 0 | 0 | 0 | 0 | 0 | 0 |
|  | Vizcaya | 0 | 216 | 0 | 0 | 0 | 0 | 0 | 0 | 0 | 0 | 0 |
| Principado de Asturias | Asturias | 0 | 865 | 0 | 0 | 0 | 0 | 0 | 0 | 0 | 0 | 0 |
| Región de Murcia | Murcia | 0 | 0 | 0 | 0 | 0 | 0 | 0 | 0 | 0 | 0 | 0 |
| **Total** | | **7914** | **3009** | **423** | **78** | **7** | **4** | **4** | **13** | **9** | **18** | **7** |
