# Supplementary figures and images for: A model for the assessment of bluetongue virus serotype 1 persistence in Spain
Source: PLoS One. 2020 Apr 30;15(4):e0232534. doi: 10.1371/journal.pone.0232534 (PMC7192634; doi:10.1371/journal.pone.0232534)

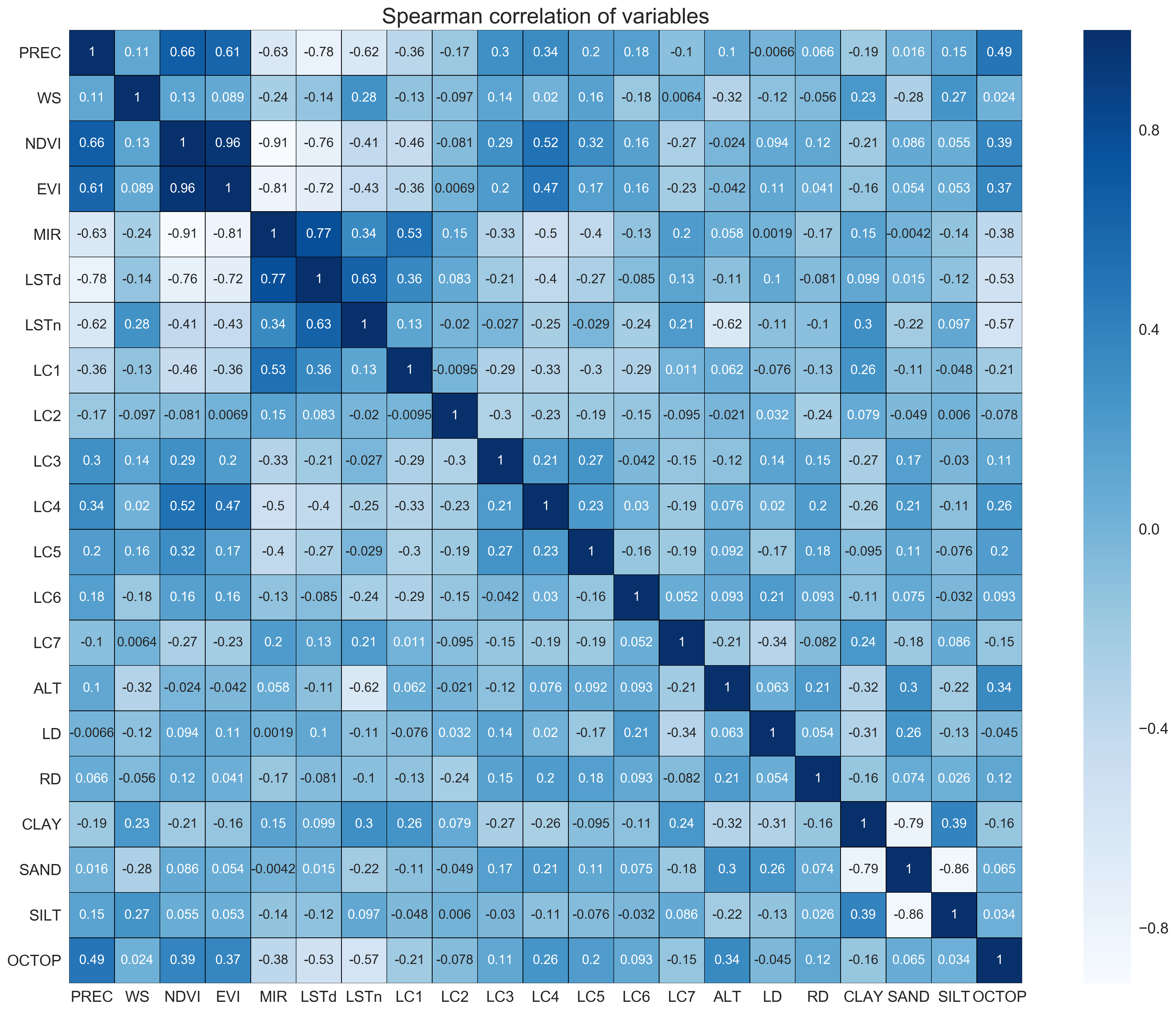

Supplement: S1 Fig — (TIF) [file pone.0232534.s004.tif]

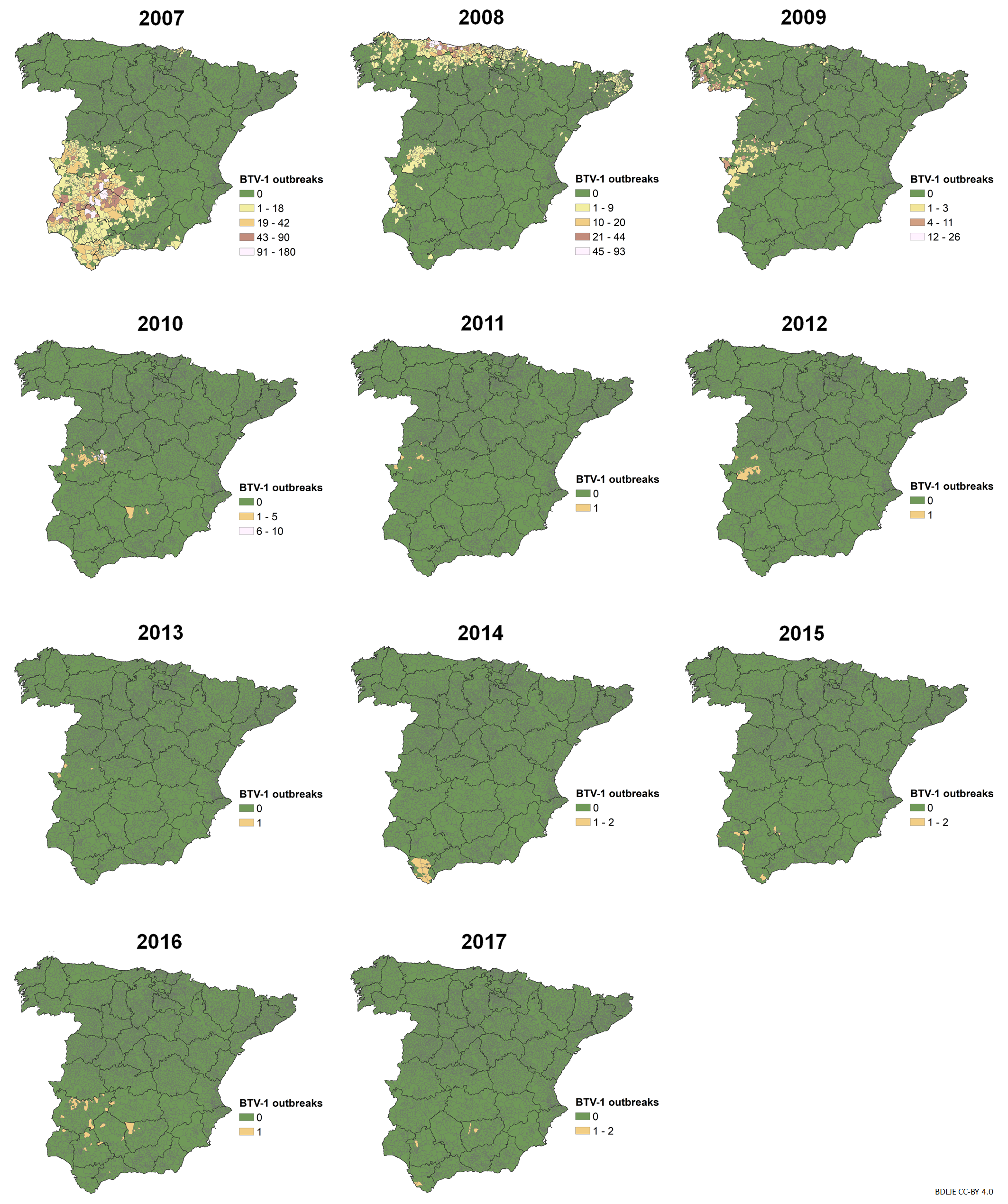

Supplement: S2 Fig — Darker lines in the map delineate provinces. Administrative boundaries provided by Instituto Geográfico Nacional (IGN); BDDAE CC-BY 4.0. (TIF) [file pone.0232534.s005.tif]

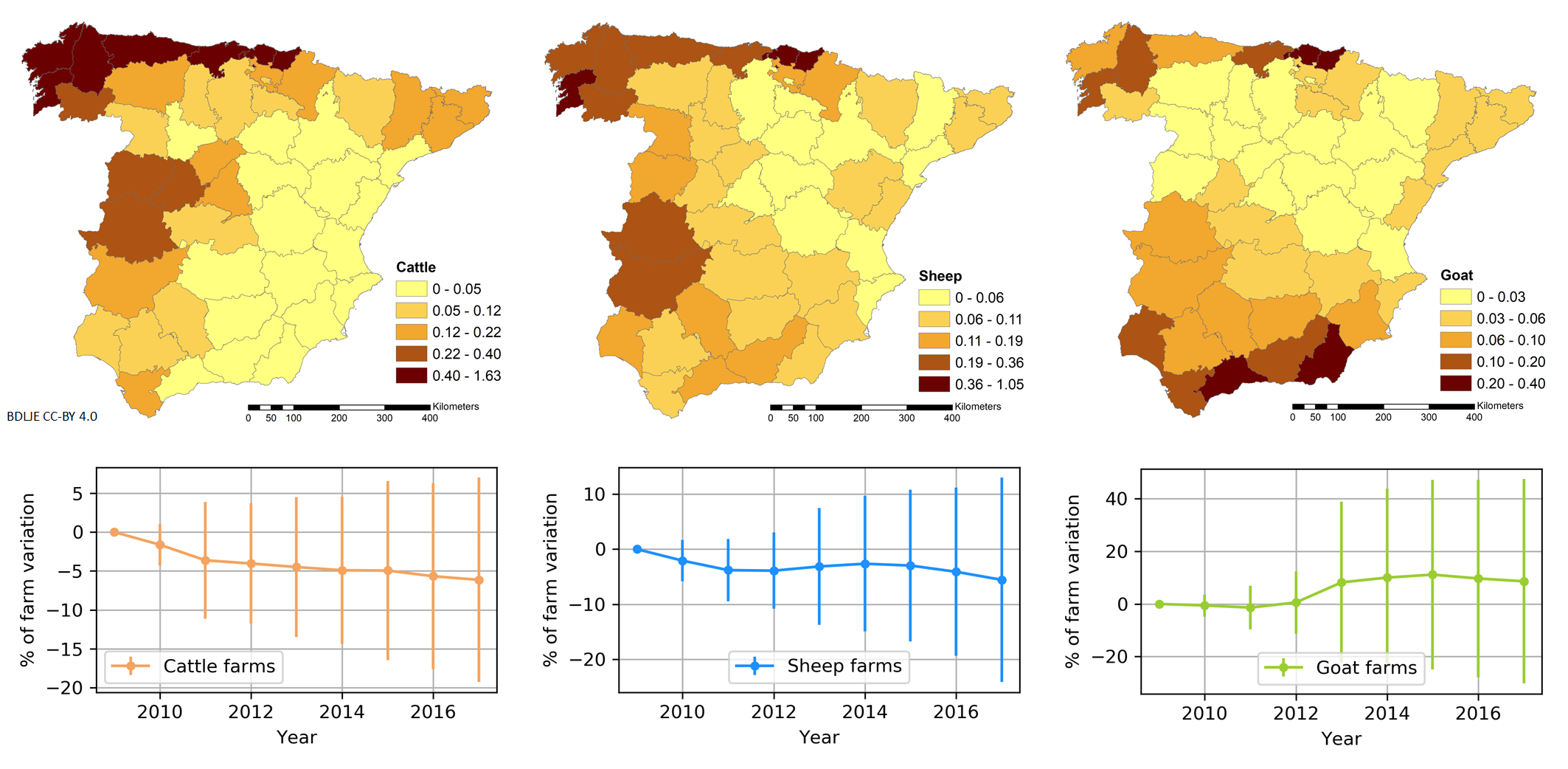

Supplement: S3 Fig — Graphs below represent the mean percentage of variation of the number of farms in Spain on the basis of the 2009 density of livestock farms; vertical lines represent the standard deviation. Administrative boundaries provided by Instituto Geográfico Nacional (IGN); BDDAE CC-BY 4.0. (TIF) [file pone.0232534.s006.tif]

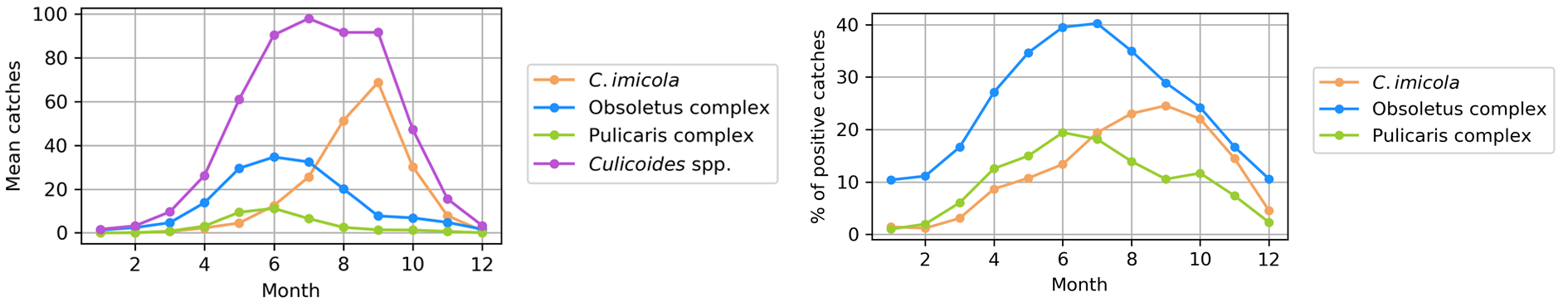

Supplement: S4 Fig — (TIF) [file pone.0232534.s007.tif]
